# Management and outcomes of vestibular anorectal malformations in a low-income country hospital

**DOI:** 10.11604/pamj.2024.47.121.42919

**Published:** 2024-03-18

**Authors:** Mahumaneng Esther Mokaila, Matlou Ernest Mabitsela, Nyaweleni Tshifularo

**Affiliations:** 1 Department of Paediatric Surgery, Sefako Makgatho Health Sciences University, Pretoria, Gauteng, South Africa,; 2 Department of General Surgery, Sefako Makgatho Health Sciences University, Pretoria, Gauteng, South Africa

**Keywords:** Anorectal malformation, vestibular fistula, anal strictures, complications, management

## Abstract

**Introduction:**

anorectal malformations (ARM) are among the most common congenital anomalies in pediatric surgery. Early detection and management of vestibular fistulas are crucial for optimal outcomes, capitalizing on the pliability of sphincter muscles and the preservation of somatosensory integration. This study aimed to assess the incidence, clinical presentation, and management outcomes of vestibular fistula ARM in a low-income hospital setting.

**Methods:**

a retrospective audit was conducted on female pediatric patients aged up to 12 years treated for vestibular fistula ARM from January 1, 2011, to June 30, 2016. Data were collected from medical records, and patients were categorized into one of three surgical management groups. Clinical assessments, preoperative procedures, and surgical interventions were meticulously documented.

**Results:**

among 656 neonates, the incidence of vestibular fistula ARM was 8.2%. Patients presented at various ages, with 69.4% being early presenters. Notably, 11.1% of cases presented after 30 weeks of age. Functional fistula, constipation, and bowel obstruction were common presenting symptoms. Associated anomalies were relatively low. The choice of surgical approach varied, with a predominant 3-stage at 68%. Complication development did not significantly differ between surgical groups (p-value 0.083). Immediate postoperative complications were minimal, but complications at definitive anoplasty varied among the surgical groups. Anal strictures and fistula recurrence were noted. At 12 months post-surgery, anal strictures persisted in 9 participants.

**Conclusion:**

this study highlights the challenges and outcomes associated with vestibular fistula ARM in a resource-constrained setting. The 3-stage approach, despite its historical preference, demonstrated suboptimal outcomes. A 2-stage procedure appears to offer a balanced alternative, particularly suitable for low-income healthcare systems. Further research and collaborative efforts are essential to refine the management of vestibular fistula ARM and improve patient outcomes.

## Introduction

Anorectal malformations (ARM) rank among the most prevalent congenital anomalies encountered in the realm of paediatric surgery. These malformations exhibit a global prevalence, presenting a vast array of clinical manifestations that span from relatively straightforward cases involving low anomalies featuring a perineal fistula to more intricate high anomalies necessitating complex management approaches [1]. Notably, among the female population, vestibular fistula anorectal malformations represent the most observed condition. Early detection and appropriate management of vestibular fistula anorectal malformations are paramount to achieving favourable outcomes, especially during the developmental stages when sphincter muscles are pliable and amenable to intervention. During this crucial window, interventions can facilitate the integration of somatosensory input, a capacity that tends to diminish with advancing age [2].

The primary objective of this study is to meticulously document the incidence, clinical presentations, and management outcomes of anorectal anomalies with vestibular fistulas within the context of a low-income hospital. By shedding light on the specific challenges and successes encountered in a resource-constrained setting, this research aims to contribute valuable insights to the broader understanding of vestibular fistula ARM, with implications for improved clinical practice and patient care.

## Methods

**Study population:** this retrospective audit focused on pediatric female patients, from birth up to 12 years of age, who underwent evaluation and management for vestibular fistula anorectal malformation within the period spanning from January 1, 2011, to June 30, 2016. Permission to conduct the study was obtained from the hospital clinical manager, and ethical approvals were also obtained from Sefako Makgatho University Research Ethics Committee (SMUREC/M/113/2016: PG). Patient confidentiality and anonymity were meticulously upheld throughout the study.

**Patient selection:** the study encompassed female patients who were either born in our hospital or referred from lower-level healthcare facilities. Referred patients typically presented with delayed symptoms, which often included distal bowel obstruction and sepsis. The decision for early or delayed management hinged on patient stability, defined by the absence of life-threatening congenital anomalies, bowel obstruction, and extreme prematurity. Unstable patients underwent an emergency diverting colostomy, followed by a multi-staged management protocol. Stable patients were selected for distinct procedures based on the preferences of attending surgeons.

**Data collection:** data for this analysis were meticulously extracted from patients' medical records and recorded in a specially designed data collection form.

**Preoperative procedures:** prior to surgical intervention, all patients underwent comprehensive clinical assessments to rule out additional anomalies such as vertebral defects, anal atresia, cardiac defects, trachea-esophageal fistula, renal anomalies, and limb abnormalities, collectively referred to as the VACTREL association. This assessment included whole-baby plain X-rays (baby gram), abdominal ultrasonography, and echocardiograms. Late-presenting patients (aged <10 weeks) also underwent abdominal X-rays to assess for a dilated rectosigmoid colon. All anorectal malformation (ARM) fistulas were preoperatively dilated using Hegar dilators (up to Hegar dilator 10) to decompress the distal bowel. Preoperative dilation was carried out for one week before anoplasty, and patients received prophylactic antibiotics for one week prior to surgery. Late presenters (aged >10 weeks) underwent preoperative bowel washouts. No bowel preparation was required for the one-stage procedure.

**Surgical management plans:** patients were classified into one of three surgical management groups: (1) one-stage group: this group underwent Posterior Sagittal Anorectoplasty (PSARP) without the need for a protective colostomy. Parenteral nutrition was administered for one week postoperatively; (2) two-stage group: patients in this group initially received a colostomy and PSARP, with subsequent stoma closure after twelve (12) weeks; (3) three-stage group: this group underwent diverting colostomy followed by PSARP, with colostomy closure occurring between 12 to 16 weeks post-diversion. All surgical procedures were performed by senior pediatric surgeons.

**Outcome assessment:** follow-up assessments of surgical outcomes were conducted regularly until patients achieved toilet training. Outcome variables encompassed age at diagnosis, presenting symptoms, age at the first procedure, type of surgical procedures, and a comparison of complications at both early (post-operation) and late (4-week follow-up), six months follow-up, and lastly, outcomes at 12 months follow-up.

**Data analysis:** demographics and clinical characteristics were summarized using descriptive statistics. Continuous variables such as age at presentation were described by mean, standard deviation, minimum, and maximum values. Categorical variables, including presenting symptoms, were summarized using frequency counts and percentage calculations. Adverse outcomes and complications of operative techniques were presented with percentages of occurrence, along with 95% confidence intervals. Cross-tabulation was performed to assess the association between the three surgical procedures and complications and outcomes. Statistical analysis was carried out using IBM SPSS version 26 running on a personal computer, with significance testing set at a two-sided alpha level of 0.05 (5%).

## Results

**Incidence of vestibular fistula anorectal malformation:** among a total of 656 neonates treated in the unit over a 5.5-year study period, 54 cases of vestibular fistula anorectal malformation were identified, resulting in an incidence rate of 8.2%. Of these cases, 35 medical files were retrieved and included in the audit.

**Figure 1:**
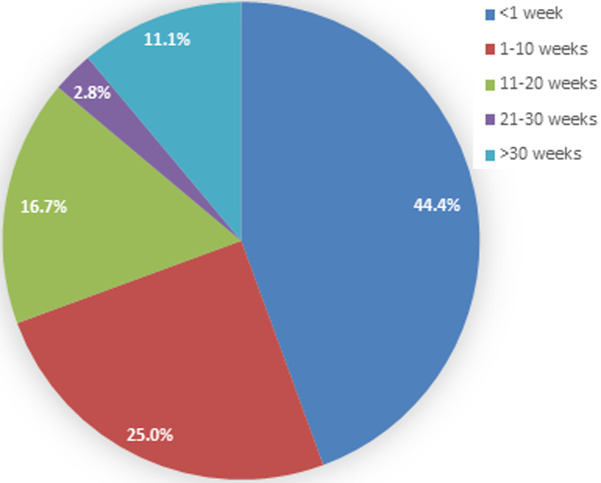
age distribution of the children at the time of presentation

**Presenting age:** the majority of patients (44.4%) presented at less than one week of age, followed by 25.0% of children between 1 and 10 weeks old. Four children (11.1%) presented at an age older than 30 weeks (Figure 1). The median age at presentation was 4.0 (13-0.1) weeks, with a range spanning from 0.1 to 156 weeks.

**Figure 2:**
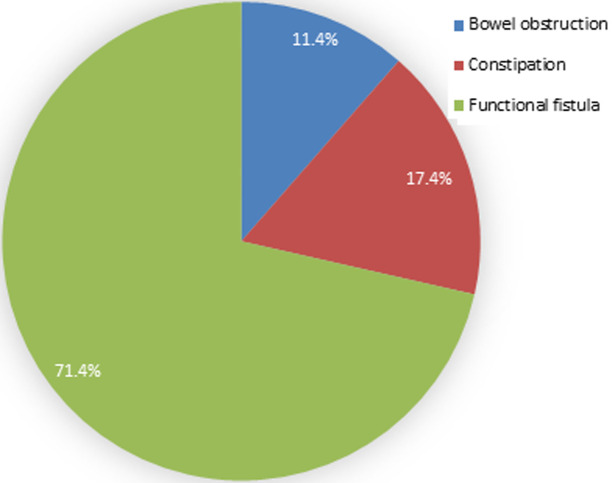
participants presenting symptoms

**Symptoms:** Figure 2 illustrates that the predominant presenting symptom was a functional fistula (71.43%), followed by constipation (17.14%) and bowel obstruction (11.43%).

**Figure 3:**
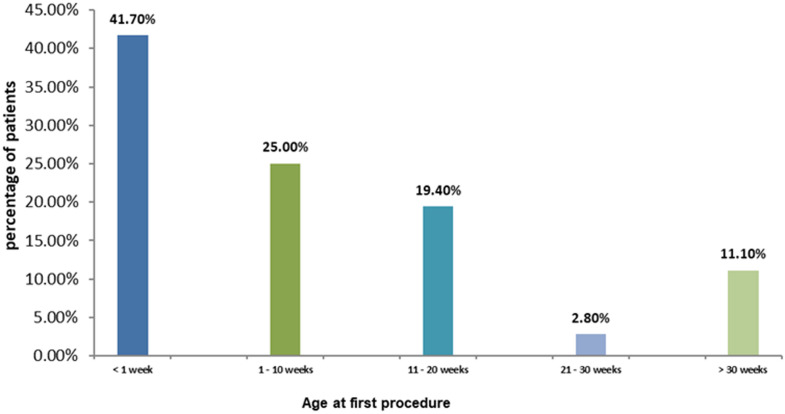
ages of the children at first procedure to correct malformation

**Age at first procedure:** similar to the age at presentation, 41.7% of children underwent their first procedure at less than one week of age, while 25.0% had their first procedure between 1 and 10 weeks of age. Four children (11.1%) had their first procedure after 30 weeks of age. The median age at the first procedure was 4.0 (13-0.4) weeks, ranging from 0.1 to 156 weeks (Figure 3).

**Figure 4:**
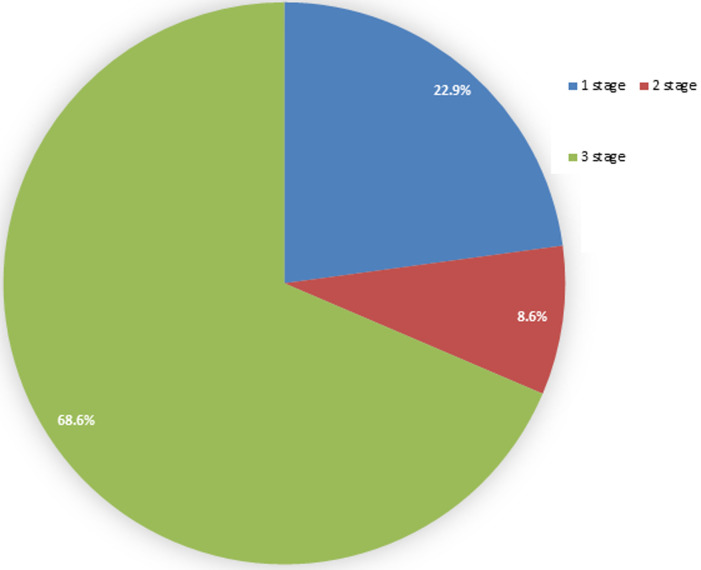
staged-procedures performed

**Complications:** in terms of the surgical management groups, 68% of the patients were in the 3-stage group, 9% in the 2-stage group, and 23% in the 1-stage group (Figure 4). The complications arising from the management of the condition in these children include (Table 1).

**Table 1:** patients outcomes by type of stage procedure

	Stage procedure			
	One stage	Two stage	Three stage	P-value
**Post-op complications**				
Stoma site breakdown	0 (0.0%)	0 (0.0%)	1 (100.00%)	1.000
Well	8 (24.24%))	3 (9.09%)	22 (66.67%)	
Defaulted	0 (0.0%)	0 (0.0%)	1 (100.00%)	
**One month follow-up**				
Wound infection	4 (66.67%)	0 (0.0%)	2 (33.33%)	0.083
Well	4 (15.38%)	3 (11.54%)	19 (73.08%)	
Defaulted	0 (0.0%)	0 (0.0%)	3 (100.00)	
**Three months follow-up**				
Anal stricture	8 (100.00%)	0 (0.0%)	0 (0.0%)	0.000
Colostomy closure	0 (0.00%)	3 (13.64%)	19 (86.36%)	
Defaulted	0 (0.0%)	0 (0.0%)	3 (100.00)	
**Six months follow-up**				
Anal stricture	3 (37.50%)	0 (0.00%)	5 (62.50%)	0.821
Persistent fistula	0 (0.00%)	0 (0.00%)	3 (100.00%)	
Defaulted	1 (20.00%)	0 (0.00%)	4 (80.00%)	
Well	4 (21.05%)	3 (15.75%)	12 (63.16%)	
**Twelve months follow-up**				
Anal stricture	4 (44.44%)	0 (0.00%)	5 (55.56%)	0.406
Persistent fistula	0 (0.00%)	0 (0.00%)	3 (100.00%)	
Defaulted	2 (28.57%)	0 (0.00%)	5 (71.43%)	
Toilet training	2 (12.50%)	3 (18.75%)	11 (68.75%)	

**Immediate postoperative complications:** immediately following surgery, only one case (4%) in the 3-stage group experienced stoma site breakdown complications. Most patients in the 3-stage group (66.67%) were well compared to those in the 2-stage (9.09%) and 1-stage (24.24%) groups. No statistically significant differences were observed among these groups (p-value 1.000).

**Complications at definitive anoplasty:** at the 1-month follow-up, six participants presented with minor wound infections, with 66.67% in the 1-stage group and 33.33% in the 3-stage group. No complications were observed in the 2-stage group, but three participants (100%) in the 3-stage group defaulted follow-up assessment. No statistically significant differences were found among the groups (p-value 0.083). At the 3-month follow-up, all patients in the 1-stage group (100%) had anal strictures, while none were observed in the 2-stage and 3-stage groups. Colostomy closure occurred in 13.36% of the 2-stage group and 86.36% of the 3-stage group. Three patients (100%) in the 3-stage group defaulted to treatment, with a statistically significant difference observed among the groups (p-value 0.000).

At the 6-month follow-up, eight participants had anal strictures, with 37.5% in the 1-stage group and 62.50% in the 3-stage group. Three participants had persistent fistulas, all of whom were in the 3-stage group. Five participants were lost to follow-up, with 20.00% in the 1-stage group and 80% in the 3-stage group. All patients in the 2-stage group had stomas closed, and no anal strictures were reported. Colostomy closure rates were 21.05% in the 1-stage group, 15.75% in the 2-stage group, and 63.16% in the 3-stage group. No statistically significant differences were found among the groups (p-value 0.821).

At the 12-month follow-up post-surgery, nine participants had anal strictures, with 44.44% in the 1-stage group and 55.56% in the 3-stage group. Three participants had persistent fistulas, all of whom were in the 3-stage group. Seven participants defaulted, with 28.57% in the 1-stage group and 71.43% in the 3-stage group. Seventeen participants were ready for toilet training, including 12.50% in the 1-stage group, 18.75% in the 2-stage group, and 68.75% in the 3-stage group. No statistically significant differences were observed among the groups (p-value 0.406).

## Discussion

**Incidence of Anorectal Malformations (ARMs):** ARMs constitute a relatively common congenital anomaly encountered in pediatric surgery, with reported incidence rates varying between 1 in 2000 and 1 in 5000 live births globally [1]. Internationally, the incidence of ARM is approximately 1 in 5000 neonates, but variations exist across geographical regions [3]. Notably, our study revealed a higher incidence rate of 8.2%, a figure that surpasses most reported rates. This heightened incidence in our setting underscores the significance of further investigation into the prevalence and management of vestibular fistula ARM.

**Age at presentation:** early diagnosis of ARM is pivotal for optimal management and favorable outcomes. Delayed diagnosis can be a concern, as Turowski *et al*. reported that even with routine physical examinations, one in five neonates with imperforated anus experienced delayed diagnosis [4]. Our study observed a wide range of ages at presentation, from less than one week to over 30 weeks, with a median age of 4 weeks. Early presenters, constituting the majority (69.4%) of our patients, underscore the need for heightened awareness among healthcare providers and caregivers to facilitate early detection and intervention.

**Presenting symptoms:** functional fistula, constipation, and bowel obstruction were the primary presenting symptoms in our patient cohort. These findings align with those reported in the literature, emphasizing the importance of recognizing these common clinical presentations for timely diagnosis and management [5].

**Associated anomalies:** although we did not observe a high prevalence of associated anomalies in our study cohort, it is noteworthy that VACTERL association, which includes renal anomalies and cardiac defects, is reported in about 10% of patients with vestibular fistula ARM [6]. Our findings align with the practice of conducting echocardiograms and renal ultrasounds during initial presentations, as abnormalities may not be immediately apparent.

**Surgical management trends:** the choice of surgical management for vestibular fistula ARM has evolved over time. While single-stage repair is gaining favor in high-income countries due to its cost-effectiveness and reduced hospital stays [7,8], there remains a persistent debate regarding the ideal approach. Traditionally, colostomy was recommended to mitigate complications such as wound infection and dehiscence. However, colostomies themselves carry significant morbidity and financial burden, especially in resource-limited settings [9-11]. Our study predominantly employed a 3-stage approach due to concerns about sepsis, wound healing, and the potential risk of future fecal incontinence. Patients offered a single-stage procedure were typically less than one week old, a strategy supported by the relatively sterile meconium and lower risk of surgical site infection in neonates [7].

**Complications and outcomes:** our study yielded varying complication rates at different stages of management. Immediate postoperative complications were minimal, with only one case of stoma site breakdown in the 3-stage group. At the 1-month follow-up, minor anal sepsis was more prevalent in the 1-stage group, possibly due to early dilatation. However, these findings underscore the importance of proper patient and parental education on home care.

At the 3-month follow-up, anal strictures were more common in the 1-stage group, potentially due to poor compliance with dilatation, as parents often considered their children's ability to pass stool as a sign of success. The recurrence of fistula in the 3-stage group raised concerns about the adequacy of fistula mobilization during PSARP, highlighting the significance of meticulous surgical technique [9,10,12]. The 12-month assessment post-surgery revealed a mixed outcome, with anal strictures, persistent fistulas, and defaults in follow-up. The high incidence of anal strictures in the 1-stage group, coupled with complications in the 3-stage group, raised concerns about the appropriateness of our chosen management approach.

## Conclusion

Our study adds to the growing body of literature on vestibular fistula ARM management. Despite a high incidence rate, we observed that the 3-stage approach had suboptimal outcomes, including anal strictures and defaults in follow-up. While single-stage repair is favoured in many high-income countries, the choice of surgical approach should consider local context, resource availability, and patient factors. Based on our findings, a 2-stage procedure appears to offer a balanced approach that minimizes complications and is well-suited to our low-income healthcare setting. Continued research and experience-sharing will further refine the approach to managing vestibular fistula ARM, with a focus on improving long-term outcomes and patient quality of life.

### What is known about this topic

- *Anorectal malformations (ARMs) are common congenital anomalies in pediatric surgery, with a variety of clinical presentations;*
- *Early detection and intervention for vestibular fistula ARM are critical for optimal outcomes, particularly during the developing stages;*
- *Recognizing common symptoms, such as functional fistula, constipation, and intestinal obstruction, is critical for timely diagnosis and intervention*.

### What this study adds

- *This study sheds light on the challenges and outcomes of managing vestibular fistula ARM in a resource-constrained setting;*
- *The study contributes to the ongoing discourse on surgical management approaches for vestibular fistula ARM by advocating for a 2-stage procedure in resource-constrained settings*.
